# Quality criteria of nature-based interventions in healthcare facilities: a scoping review

**DOI:** 10.3389/fpubh.2023.1327108

**Published:** 2024-01-11

**Authors:** Ann Sterckx, Ben Delbaere, Geert De Blust, Irina Spacova, Roeland Samson, Roy Remmen, Hans Keune

**Affiliations:** ^1^Chair Care and the Natural Living Environment, Department of Primary and Interdisciplinary Care, Faculty of Medicine and Health Sciences, University of Antwerp, Antwerp, Belgium; ^2^Department of Bioscience Engineering, University of Antwerp, Antwerp, Belgium; ^3^Department of Primary and Interdisciplinary Care, Faculty of Medicine and Health Sciences, University of Antwerp, Antwerp, Belgium

**Keywords:** mental health, physical health, social health, nature-based intervention, healthcare, relationship with nature, quality assessment, One Health

## Abstract

**Introduction:**

Implementing integrated nature-based interventions that simultaneously serve human health and the restoration of biodiversity in healthcare facilities is considered a promising strategy. As an emerging field of research and practice in healthcare, identification of quality criteria is necessary to support desired outcomes related to biodiversity, human health and intervention processes. This study is part of a larger research project in collaboration with the Flemish Agency of Nature and Forest in Belgium.

**Methods:**

A scoping review was conducted in accordance with the Joanna Briggs Institute methodology for scoping reviews, in PubMed, Medline, Web of Science and Scopus. A step-by-step tabular screening process was conducted to identify relevant studies and reviews of nature-based interventions, published in English between January 2005 and April 2023. A qualitative content analysis was conducted and the results were then presented to the project steering group and a panel of stakeholders for refinement.

**Results:**

After filtering on the eligibility criteria, and with focus on healthcare facilities, 14 articles were included in this study. A preliminary nature-based interventions quality framework with a set of quality indicators has been developed.

**Discussion:**

When designing integrated nature-based interventions, a needs analysis of users and the outdoor environment should be conducted. Next, the integration of a One Health and biodiversity perspective and the application of a complex intervention framework, could support the quality of the design and implementation of nature-based interventions in healthcare facilities and facilitate their assessment. In future work, more rigorous research into the design and implementation of integrated nature-based interventions is needed to test and refine the quality criteria in practice.

## 1 Introduction

People can improve their physical and mental health through contact with nature, as highlighted by intervention studies (1–7) and detailed literature reviews of qualitative and quantitative nature-based intervention studies (8–12). As a result, nature-based interventions (13) are emerging as a promising health promoting strategy (14, 15). NBI is mainly defined as follows: *“Nature-based interventions are planned, intentional activities to promote individuals’ optimal functioning, health and well-being or to enable restoration and recovery through exposure to or interaction with authentic nature or technological nature.”* (9). Furthermore, the Sustainable Development Goals (SDG) place ‘health’ at the center of the pursuit of global sustainability. However, to be successful, progress on the other SDGs, such as ‘climate action’ and ‘healthy environments’, are required (16). Furthermore, the growing evidence of the link between health and biodiversity (17–19) is receiving increasing attention in public health. For example, international professional health-oriented networks e.g., World Organization of Family Doctors (20), Clinicians for Planetary Health,^1^ Climate Psychology Alliance,^2^ World Health Organization (WHO) and scientific approaches such as One Health (21) and Planetary Health (20, 22) advocate the interdependence between human-nature-health in healthcare. Likewise, healthcare facilities are recognizing the importance of restoring the biodiversity of their surrounding natural environment and in providing guidance in nature for its health benefits to their target groups. The development of integrated NBIs becomes important, aiming to improve human health while simultaneously restoring biodiversity (14) and as such introduce a reciprocal human-nature-health relationship (19). By choosing integrated NBIs, healthcare facilities demonstrate their concern and responsibility for implementation of several SDGs, such as, for example SDG 3 (Good health and wellbeing) and SDG 13 (Climate action), In addition, it provides insights into novel approaches and how the reciprocal human-nature-health relationship can be implemented in healthcare facilities. However, integrating this reciprocal human-nature-health relationship into NBIs in healthcare facilities is an emerging and complex field (23). For example, NBIs in healthcare facilities are developed within an organizational context, involving multiple levels, such as management, multiple disciplines, healthcare professionals, patients, visitors and the neighborhood. Consequently, multiple stakeholders are involved. Furthermore, NBIs encompass different types of interventions (e.g., horticultural therapy, ecotherapy, and other nature-based therapies) and contexts in which they are implemented (e.g., hospitals, prisons, nursing homes) (8). Next, NBIs can occur in different types of nature, such as green or blue spaces or in a combination of both (12, 15). Due to the specificity of the organizational context, NBI diversity and the addition of the biodiversity restoration component, NBIs in healthcare facilities can be viewed as complex interventions (CI) (24). CI is an evaluation framework, as are Intervention mapping (25), implementation science or similar frameworks (26), which serve as a basis for CI design, implementation, evaluation and research. However, it is unclear to what extent these frameworks are applied in NBIs in healthcare facilities.

In Flanders, Belgium, numerous greening initiatives are being implemented in healthcare facilities. For instance, more than 180 healthcare and wellbeing facilities participate in the ‘Green deal for sustainable healthcare’, a governmental program that promotes the integration of nature into healthcare, alongside environmental measures (27). More specifically, a participating urban hospital, in collaboration with multiple partners (e.g., city, nature organizations, volunteers), launched a funded ‘Nature on prescription’ project, in which they combine biodiversity restoration in their surrounding natural environment and promote guidance of their patients in nature (27). However, this raises the question of how healthcare facilities can best design, implement, monitor and evaluate these complex integrated NBIs. Despite the need for evidence-based health promotion interventions, it is unclear what quality criteria healthcare facilities in NBIs use to achieve desired outcomes. Based on the previously mentioned CI, IM or similar evaluation frameworks, and the biodiversity-health link (17, 19, 28), identifying criteria for integrated NBIs could be related to health, biodiversity and intervention processes outcomes. In addition, a number of recent reviews address the implementation of NBI in healthcare (10, 29–32), but these studies have not specifically focused on the underlying quality criteria for implementing and designing integrated NBI in the surrounding natural environment of the healthcare facility. Quality criteria are crucial to support the quality of the design, implementation and evaluation of interventions or programs, and this principle also applies to integrated NBIs. For example, in terms of design, quality criteria help develop interventions that are evidence-based, efficient, and tailored to specific healthcare needs and desired outcomes. Quality criteria can guide the implementation process, and ensure compliance with established standards, protocols, and best practices, as well as promote consistency and reliability. Finally, they enable rigorous evaluation of the effectiveness and impact of interventions, while identifying areas for improvement or adjustment (24, 25).

Therefore, the aim of this study was to identify quality criteria that are relevant in the different phases of the NBI and to develop a quality assessment framework for integrated NBIs in healthcare facilities to be tested and refined in a subsequent qualitative study. The research question in this study was *‘What is known in the literature about the quality criteria of integrated nature-based interventions in healthcare facilities?’*

## 2 Methods

A scoping review was conducted in accordance with the Joanna Briggs Institute (JBI) methodology (33) in combination with the Preferred Reporting Items for systemic reviews and Meta-Analysis extension for Scoping Reviews (PRISMA-SCR) (34). The focus was on recent peer reviewed studies regarding NBI in the surrounding natural environment of healthcare facilities. Although a published protocol is available (35), a summary of the methodology is provided in what follows.

This scoping review is part of a larger research project funded by the Flemish Agency of Nature and Forest (FANF), with the aim of developing a quality assessment framework for NBIs designed and implemented in healthcare facilities. To conduct the scoping review an interdisciplinary research team within the Chair Care and the Natural Living Environment (University of Antwerp) was set up with expertise in the fields of ecology and biodiversity, nature and human health, organizational psychology and nursing. Next, a project steering group consisting of the research team, and experts in ecology of FANF, and a policy and knowledge expert of VIPA, a knowledge center of the Flemish governmental department of Wellbeing and Care, follows the progress in the research project. Finally, a panel of stakeholders (36) from different disciplines and sectors was engaged to refine the results of the analysis.

### 2.1 Search strategy

We used two approaches for our search strategy or relevant publications. First, a preliminary search for peer reviewed NBI-related reviews was conducted in four databases, PubMed, Medline, Scopus, and Web of Science, with the aim of identifying the most relevant search terms according to the NBI topic. Titles, abstracts, keywords and index terms of these reviews were screened. Second, due to the complexity and vastness of the research field of biodiversity, as well as the time constraint in which the larger research project is taking place, a set of search terms was created together with two ecology experts (GDB, RS).

#### 2.1.1 Search terms selection and refinement

All identified preliminary search terms were discussed with the research team, including the ecology experts, to refine the selection in this study. Next, an experienced librarian from the University of Antwerp, Belgium assisted in the development, testing and refining several sets of search terms. As a result, two sets of search terms were constructed, one for nature-based interventions and another one for biodiversity, which were combined in the search [for detailed search term listings see the protocol (35)].

#### 2.1.2 Eligibility criteria for studies included in the analysis (population, concept, context)

Using the identified search terms, qualitative, cross-sectional and other quantitative peer reviewed NBI studies and reviews, published in English (for pragmatic reasons) between January 2005 and April 2023, were included in the analysis. The eligibility criteria were defined according to population, concept and context, generally used in scoping reviews (33).

Studies were included when the target population were patients or healthcare staff (population) connected to a healthcare facility, and the NBI was implemented in a healthcare institutional setting (e.g., hospital, residential care facility, nursing or retirement home or alike), surrounded by green or blue space. Studies on green care farms were included only when they focused on the benefits of the natural environment on human health (concept and context).

##### Exclusion criteria

In the first and second step screening process studies were excluded when ‘wrong context’ (such as limited to animal-assisted care interventions, community gardening, focus on other objectives than healthcare (e.g., recreation), ‘no nature’ (indoor and virtual nature), no institutional healthcare setting (e.g., schools, social work, care farm with only agriculture) or target groups with no specific care needs (e.g., citizens), ‘wrong type of publication’ (individual case studies, study protocols, conference papers, background articles, books, opinion papers, editorials, position papers, commentaries), laboratory studies not situated within the design and implementation of NBI).

#### 2.1.3 Screening process for study inclusion

**Figure 1:**
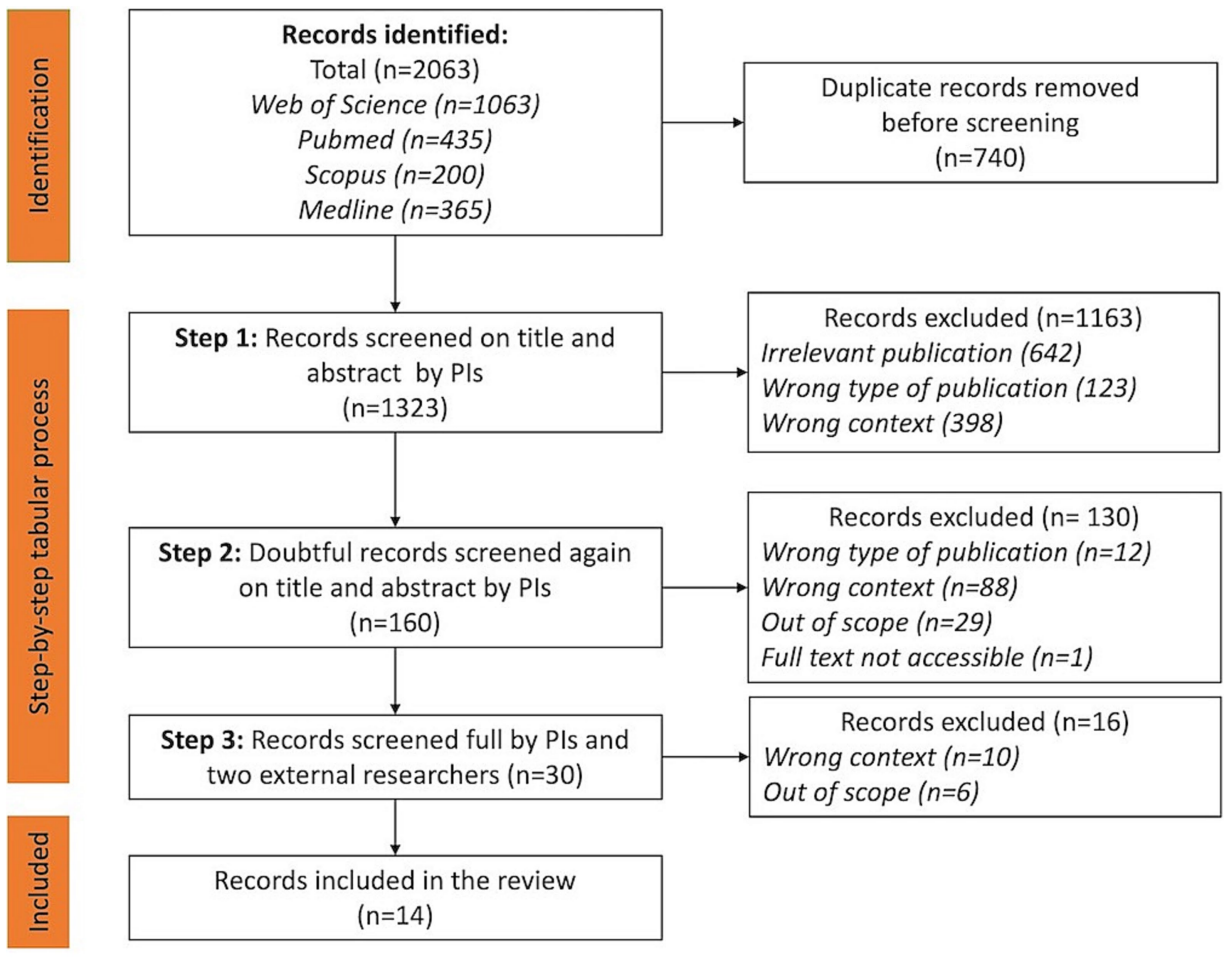
PRISMA flowchart.

A step-by-step tabular process was conducted to identify relevant NBI studies to be included. In the first step, the two principal investigators (PIs) (AS, BD) conducted an independent screening based on title and abstract for relevance as well as inclusion and exclusion criteria. The PIs made the following decisions: not relevant, doubtful, include. The second step was the joint review of the ‘doubtful’ articles by the two PIs. Potential researchers’ screening bias and disagreements were resolved through discussion, consensus, and consultation with the interdisciplinary research team. Finally, the full article texts were read by the two PIs and two other researchers. Figure 1 shows the PRISMA flowchart for inclusion of studies.

### 2.2 Data extraction

To capture the context of NBI, study-related data was first extracted from the selected publications and sorted in an Excel spreadsheet according to the following predefined fields: Author(s), year; country, type and aim of the study; type of facility; target group, type of NBI and activities, outcomes. Next, searching for quality criteria for ‘integrated NBI’, per study data was extracted regarding criteria that might be associated with biodiversity, human health and intervention processes (Supplementary Table S1). Finally, hindrances and barriers encountered in the design and implementation of NBI was noted as well.

### 2.3 Data analysis

Data analysis was carried out in two steps using a qualitative content analysis (37, 38). The first step, performed by the two PIs, concerned an analysis of the contextual characteristics of the NBI per extracted study, namely in terms of the type of NBI, the target group, the type of healthcare facility and outcomes. The second step was performed by the two PIs and two additional researchers and consisted of identifying NBI quality criteria, categorized by quality domain (human health, intervention, biodiversity). In addition, the barriers and hindrances in the design and implementation of NBIs were identified per study when mentioned in the text. Finally, the results were presented to the project steering group and the stakeholders’ assembly.

## 3 Results

A total of 1,323 publications (duplicates excluded) were found using the search strategy described above at the first step of the search. Next, after filtering on the eligibility criteria and reading the full texts, finally 14 publications (5 reviews, 1 pre and posttest design of experimental and control groups, 5 qualitative studies, 3 mixed methods), were included in this review. Four articles (13, 39–41) are discussed in the selected reviews as well. However, they were retained in this study because they each identified additional elements relevant to our study that were not discussed in the reviews.

First, we will discuss the contextual characteristics of the NBI in the included studies. Second, the findings on the quality criteria will be discussed.

### 3.1 Overview of the types of facilities, target groups, nature-based interventions, their context and outcomes in NBI

Supplementary Table S1 summarizes the contextual NBI-characteristics in each study. Here, we will discuss each characteristic separately.

#### 3.1.1 Type of facilities and target groups

When considering healthcare settings, the NBI are mainly applied, respectively, in residential care centers and target group-oriented care facilities (e.g., residents with dementia, rehabilitation) and hospitals. Specific examples include psychiatric inpatient units (41) and nursing homes (42). Target groups are mainly patients with dementia, older adult people, people with a mental or psychological frailty and rehabilitating patients. Two studies (43, 44) also pay attention to how healthcare professionals (HCP) experience self-oriented health benefits of going into nature during their work.

#### 3.1.2 Types of nature-based interventions and their context

Several types of NBI and nature-based activities are mentioned in the selected studies, using different names interchangeably. Most of the studies concern horticulture therapy (HT). HT consists of using plants and gardening (13), ranging from preparing the soil to harvesting. In addition, HT can be subdivided according to its objective: (1) focused on therapeutic walking gardens that do not require active participation from participants; (2) gardening (ranging from tillage to harvesting) in vegetable gardens at the healthcare facility; (3) gardening with commercial purpose, such as use of the cultivated crops for artistic creations, for personal use in the healthcare facility or for sale to visitors to the healthcare facilities or at local markets (8).

Next, two reviews discuss ‘green care’ (39, 45). As in HT, the term ‘green care’ is often used as an umbrella for implementing nature-based interactions and activities in nature for health purposes (45). These interactions can vary from spontaneous to organized nature-oriented activities and experiences within or outside an institutional context. In this study we focus on green care within an institutional context.

#### 3.1.3 Types of intervention outcomes

NBI are mainly used to benefit a variety of human health outcomes. In line with the definition of health proposed by World Health Organization, the discussed health outcomes in the selected studies relate to general wellbeing, mental health, physical health, social health and spirituality (46). Upon more detailed examination, the specific mental health-related outcomes included both qualitative (e.g., descriptions of improved mood) in semi-structured interviews (41) and quantitative read-outs, such as happiness and depression symptoms (44). However, reporting of quantitative physical health outcomes were in the minority, as also Moeller et al. (8) noticed in their review. Next, some studies (13, 43) suggested important recommendations regarding garden design, supporting the health outcomes of the NBI.

### 3.2 The identified quality criteria in design and implementation of nature-based interventions

In the selected studies, we found several criteria (Supplementary Table S1) that can be mainly classified into two quality domains, namely quality criteria supporting human health and intervention processes. We did not find any studies in which NBI were simultaneously designed for human health and biodiversity restoration in healthcare facilities. More specific, quality criteria for the restoration of biodiversity were missing in the selected studies.

#### 3.2.1 Quality criteria for human health

Here we report criteria likely to support human health outcomes, such as needs assessment, the quality of guidance of the target population, the role of the HCP, the structural design of the natural environment for health purposes, and the quality of nature interactions in NBIs.

##### Conducting a needs analysis: Users and outdoor environment

Several studies mention that the NBI should be designed according to the needs of the target population (e.g., patient, HCP staff, visitors) (13, 39, 41, 43). A design of an NBI may concern the program elements of the intervention (e.g., choice of activity, required guidance) and the design of the garden. Pieters et al. (41) found that NBI program elements can be modified to better meet patient needs. For example, with regard to dementia, the level of dependency of the patients impacts the intensity of guidance (44) and the type of nature that will be used (e.g., edible plants, quiet garden versus activity garden) (44). Another aspect is the importance of risk assessment (492021), such as the risk for sunburn and dehydration (47). Although some design aspects of the outdoor environment may support a certain part of the target population, they may pose a risk to others e.g., use of water features, smoking areas (44), choice of plants (43). However, it appears that many NBI are often not adopted to personal needs, abilities and preferences of the target group (13). Therefore, to optimize the NBI design, a systematic analysis on personal needs, the strengths and weaknesses of the outdoor environment in relation to these personal needs is pre-requisite (13), and a person-centered care approach (39, 42, 47) should be at the base of the NBI design. In all, a ‘user analysis’ should be performed when designing NBI. A user analysis is an analysis of the needs of the target group, in which data is collected by observation, questionnaires or interviews (43). In addition, an analysis of the outdoor environment would give insight into its possibilities and barriers for the design and implementation of the NBI (43).

##### Quality of the guidance of the target population

Several studies discuss the quality of the guidance offered by the HCP based on its intensity, duration, structure of nature-based activities, and the use of a supportive healthcare team. Overall, they can be viewed as components of person-centered care in the design and implementation of NBIs.

###### Level of intensity

Depending on the type of activity (e.g., walking, gardening, art therapy, relaxation exercises) and the health condition of the target group, there appears to be a difference in the intensity of the guidance. For example, Oh et al. (48) described the type of guidance (e.g., training, instructions, and demonstrations of gardening tasks) required for patients with a schizophrenic disorder, as well as the required counselors for guidance of the patients and HCPs for advice and supervision (48). In another study HT sessions were supervised by a certified horticulture therapist, an intern, and a HCP from the healthcare institution itself (42). Furthermore, another study mentioned that HT sessions with sensory stimulation, held in a psychiatric hospital, were guided by a therapist under the supervision of two occupational supervisors (41). In a review some NBIs consisted of using nature as a context to wander around, with occasional mention of concrete observations of nature, while in other cases, for example, relaxation exercises in nature were guided by an HCP (49). So, mostly NBIs require several HCPs to guide, advice or supervise during the implementation of the NBI.

###### Duration, frequency and total length

A variety in formulas of duration and frequency of each NBI-session, and total length of the NBI-intervention were found in the selected studies. However, it is not clear on which evidence these choices have been made. Duration of each session varied between 45 min (41), 60 min (42, 49, 50), to maximum 120 min (48). Frequency of the sessions were held weekly (42, 48) to twice a week (47). The length of the NBI-intervention lasted between minimum 8 weeks (42), 10 consecutive weeks (47) to 9 months (50).

###### A supporting healthcare team

Providing a dedicated healthcare team is valuable to prepare participants to feel comfortable before embarking on a nature-based activity (41). They can also take patients outdoors between indoor therapy sessions or they can assist their colleagues during the nature-based therapy and on site during high-risk moments (49).

##### The role of the healthcare professional

The HCP can contribute to the quality of the NBI in various ways.

For instance, HCPs, in particular occupation therapists, can use their expertise in the choice of activities and use of the environment to reach their therapeutic goals (44). Advice can also be given on the garden design to support the patients’ involvement in gardening tasks (47). Additionally, HCPs should support active engagement in nature-oriented activities, support the implementation of the NBI and ‘using it to its full potential’ (39), and be trained and competent when guiding in patients in NBI (44). For example, in HT, it seems important to mentally prepare participants for what is to come (44). A qualitative study found that the accompanying HCP’s knowledge of gardening and plants when guiding patients in the garden positively contributes to the feelings of the patient’s safety (41).

NBI-sessions with their patients can also relax the HCP themselves (49). In addition, another study, based on the responses of HCPs who garden with patients, suggests a possible preventive effect on burn-out (13). Another study found that nurses liked to visit the garden, as long as they had sufficient privacy and contact with nature (43). Overall, incorporating the natural environment during their work could have a dual health benefit for the health of the patient and the HCP, and thus potentially contribute to the prevention of illness among HCPs. Sickness leave of HCPs in healthcare is a severe problem. For example, the Flemish Employability Monitor 2019 (51) reports that before COVID-19, 13,6% of the employees suffered from burnout symptoms, of which 15,2% were reported by HCP. Therefore, NBI in healthcare facilities could also be a promising and complementary entry into illness prevention of HCPs. However, further research is needed.

##### The structural design elements of the surrounding natural environment of the healthcare facility for health purposes

The design of the surrounding natural environment, which are mainly described as gardens, usually focus on how it can provide mental, physical, and social health benefits (43) and how the natural environment can meet the needs of the target groups (13, 47). For example, Paraskevopoulo et al. (43) discussed in their review evidence-based design recommendations for healing gardens for children in pediatric hospitals, patients suffering from cancer, mental disorders, pregnancy or infant loss, and finally for healthcare staff.

The accessibility of the gardens for specific target groups seems to be one of the most frequently cited design recommendations (44, 47). The research of Hall et al. (47) also suggests using the experience of HCP and an architect to make the garden more inviting for unsupervised activities. This means that design elements that reflect the objective of the NBI should also be considered, for example, when to use strong-smelling plants, tighter designed or wilder places, type of pathways, type of greenery, the degree of opportunity to be in solitude or in community (47). In addition, it also seems important that there are sufficient seating areas, especially for people with decreased mobility (49). Another study proposes that an environment analysis based on ‘*objective, agreed and adapted criteria’* (13) should be carried out to identify the potential and weaknesses of the existing (green)space to match the target group’s capabilities (13).

Conversely, other context-specific elements (e.g., degree of sealing, collaboration with the neighborhood, hospital architecture) influence the design of the garden and the NBI. For example, a green care farm benefits from the presence of green space, gardens, and domestic animals that provide residents with a supportive environment in which to initiate activities and go outdoors (39).

##### The quality of nature interactions in nature-based interventions

Interactions with nature in NBI can take many forms, ranging from engaging in nature activities (e.g., walking, doing relaxation exercises, gardening) to stimulating the senses and enjoying the beauty of nature to experiencing existential feelings. Below we provide an overview of the nature interactions found in the selected NBI studies.

###### Activities in the natural environment or garden

Moeller’s et al. review of NBIs (8) shows that many studies generally report little detail about which activities are specifically included in the NBI counseling sessions of the target group. A study of HT describes that participants are asked to do gardening, tasks such as digging the soil, removing dead leaves, and tending plants (41). Other duties may include caring for flowers and plants, sowing and harvesting seeds. In another study, participants were selected to participate as fully as possible in the entire gardening cycle, with activities such as creating planting beds, transplanting, watering, weeding, fertilizing and harvesting (48). In general, the extent of participants’ intensive activity in nature varies, ranging from exposure to nature (e.g., sitting or wandering around) to working in the garden.

The choice of activities, especially in HT, seems to be tailored to the possibilities of the available space of the healthcare facility, and to the characteristics of the target group (8). In addition, it seems to increase engagement when participants are able to choose for themselves between the activities or gardening tasks offered whenever possible (41).

###### Esthetic and sensorial experiences

Several studies refer to sensory stimulation by the natural environment or landscaped garden, (8, 41, 43, 45, 52). Examples include the possibility to enjoy visual stimuli (41, 43, 45), smells (41, 43, 45, 47), sounds (13, 43, 45), tactile interactions with plants (41, 43–45, 47, 50) and beauty (8, 43, 52). One study speaks about ‘sensory gardens” (52), in which sensations and beauty can be experienced with different senses, such as esthetically experiencing of colors and smells, and touching the plants and the soil (8, 13, 45). Another study refers to gardens with such sensory qualities as ‘healing gardens’ (43).

###### The mirror of nature

Another possibility is that nature can act as a mirror, in which the participant can reflect on metaphors presented through the interaction with natural scenes or nature experiences. For example, the caring aspect in gardening can be a mirror for taking care of oneself (41, 50).

###### Existential and ecological experiences, such as ‘connection with life’

In a literature review of qualitative studies, a study describes the garden adjacent to residential care homes *“as a place where connection can be made with life”* (52). This finding was based on the following experience of the participants: ‘*being able to have close contact with their self, contact with others, organized or not; connection with nature by being able to experience the seasons and by doing activities in the garden. In addition, the garden was also described as a place of experiences, through sensory stimulation and beauty experiences*.’ Many older people also experienced the garden as a place where one could feel healthy and alive. In some cases, the presence of a garden also influenced the choice of healthcare facility. Finally, the garden was also seen as a place where past and present come together, by revisiting past memories of nature, and as an opportunity to break a daily routine by being able to visit or work in the garden. Based on the findings of their research, they speak of ‘human flourishing in dignity’, and this through the inclusion of and contact with nature in healthcare facilities (52). A qualitative study in a psychiatric ward found that patients found it important to care and cultivate plants, as a metaphor for their own healing (41).

#### 3.2.2 Intervention processes quality criteria

Intervention processes can be supported by several quality criteria, such as the establishment of a project group, the use of theoretical and evidence-based frameworks, adopting a multi-layered approach, and the role of the HCP (e.g., competencies, relationship with nature).

##### Establishing a multidisciplinary project group

In the study of Jonveaux et al. (13), the project group consisted of physicians, nurses, and a psychologist for determining health goals. In the same NBI-design, a larger workgroup was then set up, consisting of the project group enlarged with landscape gardeners, engineers, technicians, and communications service specialists. James et al. (49) found that having a multidisciplinary support team was not only important for the design and implementation of the NBI, but also to assess the risks, in which HCP and occupation therapists should be consulted. On the other hand, a systematic literature review on healing hospital gardens, found that only five gardens out of 13 cases were designed by a project group, design team or architect (43), which suggests that appointing a project group or similar is not always a common practice. Even when designed by an architect, this is far from what a project group can do.

##### The use of theoretical and evidence-based frameworks

Part of scientific underpinning NBI lies in starting from scientific frameworks (24, 25, 53). However, the included studies pay limited attention to the scientific frameworks used in the design of the NBI. They mainly relate to scientific frameworks for positioning their own research, but only to a limited extent, and as such they are not perceived as a starting point to design the NBI. Nonetheless, some sources refer to Kaplan’s Attention Restoration Theory (41, 45, 49), Ulrich’s Stress Reduction Theory (41, 49) and E. O. Wilson’s Biophilia Hypothesis (45, 49). Another example is the use of health geography, which assumes that the experience of place and health are linked (52).

### 3.3 Barriers and obstacles in design and implementation of NBI in healthcare facilities

Knowing the barriers and obstacles in the design and implementation of NBI can inform the quality criteria we are looking for. Several barriers were mentioned located at the level of the NBI participant, staff level, the healthcare facility, and the design of the garden.

Several barriers were raised at the level of the participant. *Lack of training or education in gardening* of the participant may cause difficulties in the implementation of therapy (8). Next, some things have been said about *weather conditions*. For example, in Moeller’s review (8) one study found that when the weather conditions become worse, conflicts may occur between patients. Another point was that ensuring physical safety in poor weather conditions may also hinder visiting nature (49). Furthermore, the garden can be perceived as a space with *an increased risk of falls* and other safety issues, hindering the target group to participate in the NBI (42, 44, 49). Also, the *lack of programs or activities* that encourage use of the gardens by patients and visitors (43) can hinder the success of participation to an NBI. Finally, *self-image of being too old or lack of confidence* were mentioned as a possible barrier to participate in an NBI (42, 45). In addition, *unwanted sensory stimulation* resulting in fatigue by the patient might be a barrier as well (47).

Furthermore, sometimes staff can show negative attitudes and perceptions toward NBI (45). For example, participants experienced that the HCP did not talk about HT outside the outdoor sessions (42). Next, healthcare facilities suffer from limited staff time to supervise residents (45). Finally, the informal setting of guidance in a natural environment can hinder the therapeutic relationship, where the patient might infer that the relationship can become more amicable (49).

At the level of the facility limited resources to organize NBI can be a barrier for its implementation (45). Also, the lack of person-centered care culture can jeopardize the NBI (45). In addition, NBI cannot always be tailored to certain target groups. What is positive for one target group may be inconvenient or risky for another, introducing an element of complexity in the NBI-design. For example, one therapeutic approach or garden design does not fit all types of dementia (44). Finally, poor accessibility garden design (e.g., poor accessibility and safety, lack of outdoor rest areas, lack of specific recognition or landmarks) was also mentioned (45).

## 4 Discussion

The aim of this scoping review was to identify quality criteria that could be of use in the design and implementation of integrated nature-based interventions in healthcare facilities.

**Figure 2:**
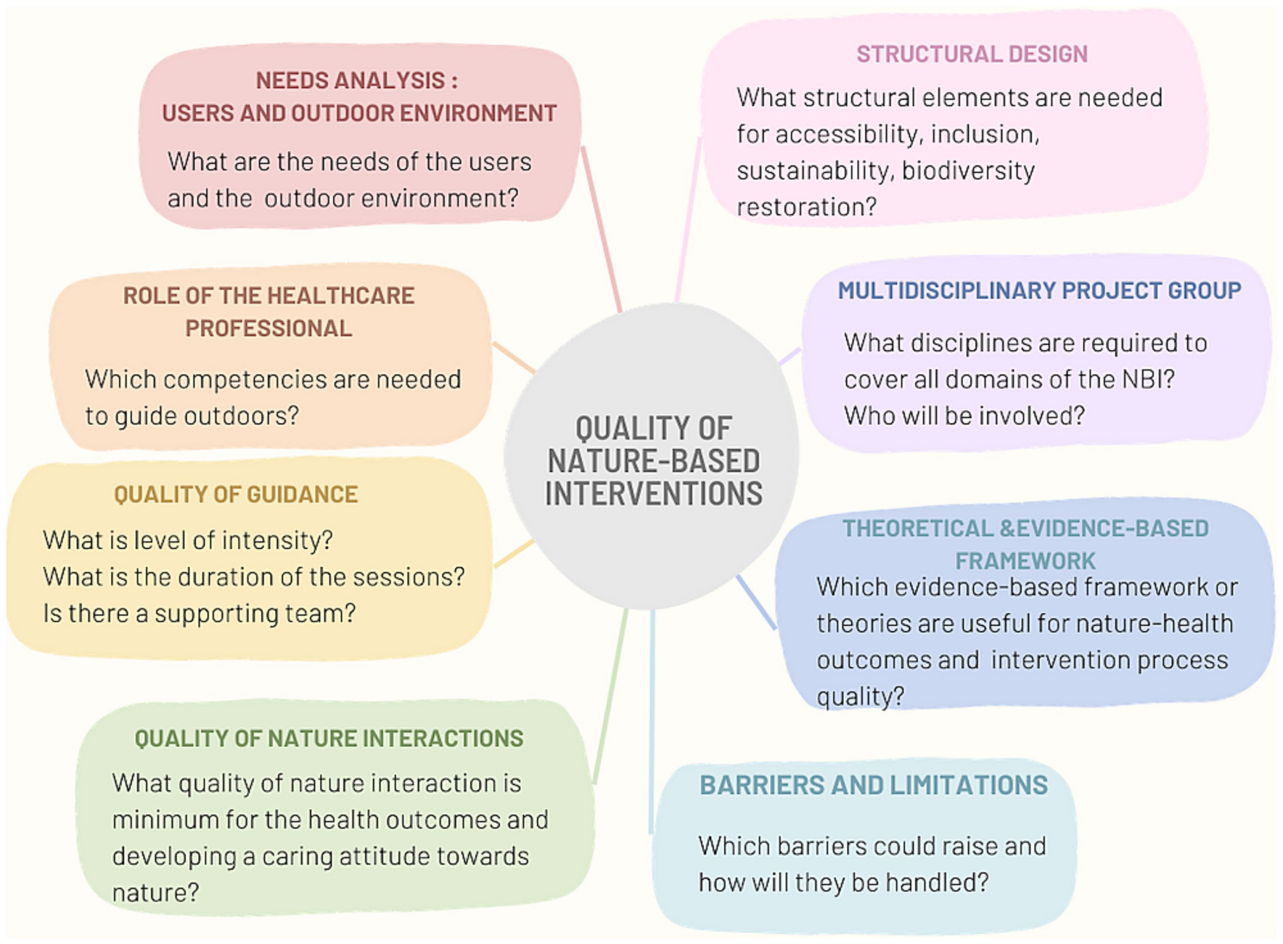
Overview of the preliminary framework of NBI-quality criteria for human health outcomes and intervention processes.

**Figure 3:**
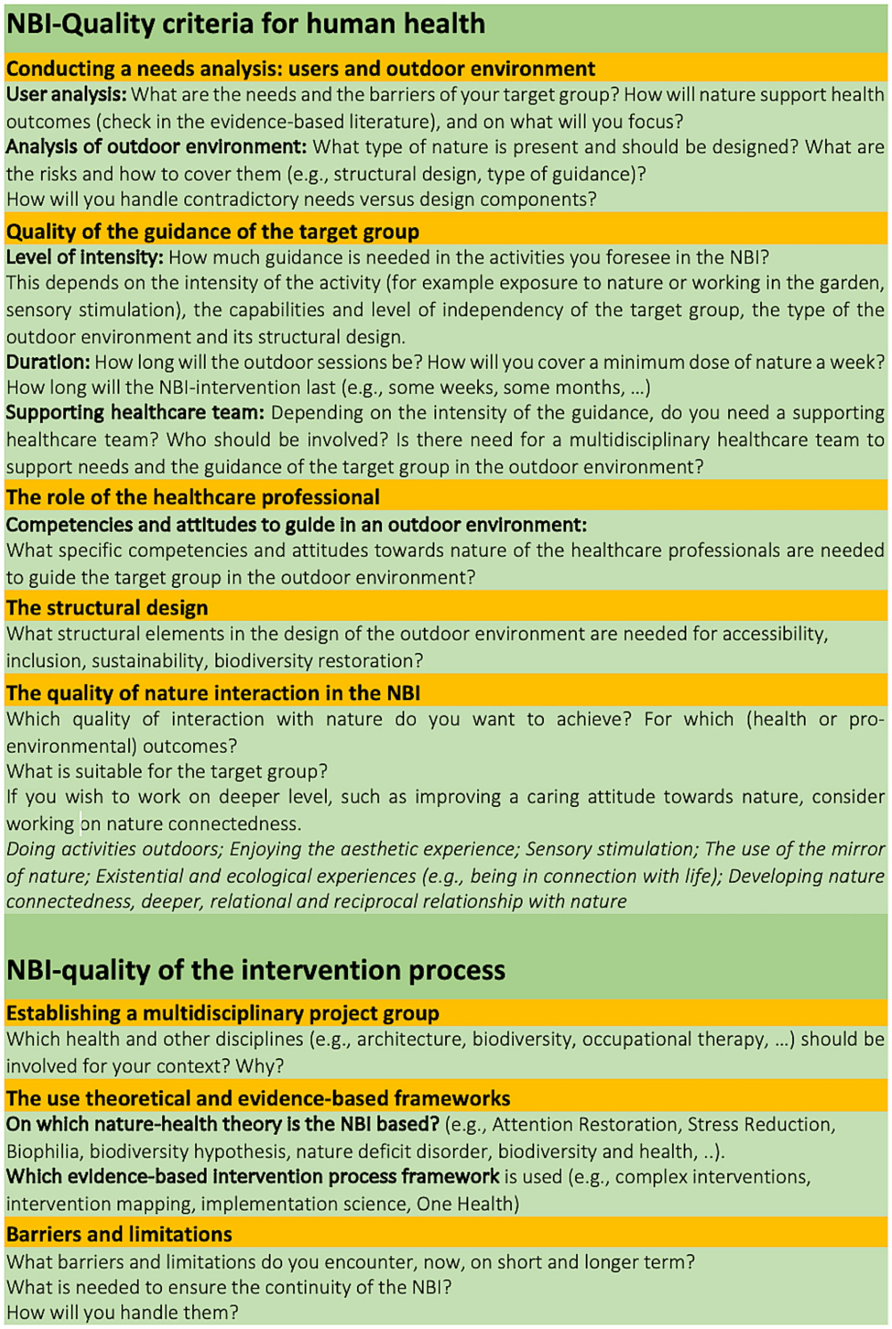
Detail of the preliminary framework of NBI-quality criteria for human health outcomes and for intervention processes.

Our study resulted in an overview of several identified quality criteria to be considered when designing and implementing NBI in healthcare facilities (Figure 2) and led us to a preliminary NBI-quality criteria framework (Figure 3). The use of quality criteria supports the development of equitable, safe and adaptive protocols during implementation, as observed in studies proposing NBIs in an organizational setting (54).

Quality criteria to support positive human health outcomes were mainly associated with person-centered care and detailed in conducting a needs analysis of the target group and the surrounding natural environment, the intensity of the guidance, the duration of the sessions, the structural design of the natural environment, the role of the healthcare professional and the quality of the interactions with nature in the NBI activities and guidance. Furthermore, quality criteria supporting the quality of the intervention processes were identified as well: establishing a multidisciplinary project group, the use of theoretical and evidence-based frameworks and research, and the role of the healthcare professional in terms of competencies. Our study revealed also potential barriers and obstacles during the design and implementation of NBIs. Furthermore, the role of biodiversity around healthcare facilities as a determinant of both nature and human health is overlooked in the reviewed studies. Consequently, there is a research gap in combining the restoration of biodiversity and guiding target groups in the surrounding natural environment of healthcare facilities.

The identified NBI quality criteria offer several advantages. They ensure evidence-based interventions, which are essential in healthcare facilities in this relatively new field of research and practice. Through person-centered care, they enable the customization of NBIs for patients, staff, and visitors. In addition, the variety of qualities of interactions with nature makes it possible to design and implement the NBI in a context specific and target group-oriented manner. However, given the findings, several additional aspects need to be considered when designing integrated NBIs in healthcare facilities.

### 4.1 NBI from the perspective of complex interventions

The human-nature-health interlinkages are a complex issue, and NBI can therefore be viewed as complex interventions (CI). Considering person-centered care and the needs for restoring biodiversity nearby the healthcare facility, while also considering their interlinkages, appears to be an important, but complex aspect of NBI design and implementation. Therefore, it is surprising that we do not see clearly articulated CI or intervention mapping (IM) frameworks, or alike, as they support and assess the targeted health interventions outcomes. CI and IM promote to use theoretical and evidence-based frameworks to inform and design a robust NBI.

### 4.2 The use of a person-centered care framework in nature-based interventions

The person-centered care framework (PCC) (55) offers opportunities for the quality framework of integrated NBIs, detailed in what follows.

The PCC framework encompasses a holistic approach to healthcare where the patient should be viewed as a ‘whole being’, with their personal beliefs, needs and preferences, above their medical needs and as such contributes to the person’s quality of life (55). In this sense, their emotional, social and spiritual well-being is taken into account, an aspect that we also encounter in the desired outcomes of NBI. In addition, we suggest questioning the person’s experience and attitude toward nature to include their preferences and beliefs on this aspect. NBIs should also consider the aspect of diversity and inclusion and promote sensitivity to race, ethnicity, gender, sexual identity, religion, age, socio-economic status and disability (55, 56). Furthermore, the PCC framework suggests to co-design a framework for monitoring, measurement and evaluation of the healthcare intervention (55). Another opportunity presented by this PCC framework is when designing and implementing NBI, criteria should be defined on each level of the organization [based on the PCC framework of Santana et al. (55)]. For example, on the level of the organization, the following could be considered: the organizational culture to promote the implementation of NBI, the establishment of a dedicated multidisciplinary project team, and the creation of frameworks to monitor the outcomes of the NBI. At the HCP level it could be about involving them into the co-design of the NBI, develop engaging communication about the NBI, deciding on the type of guidance, and the access to nature in the NBI. At the level of the patients, the HCP could listen deeply to the preferences and needs regarding their relationship with nature, their preferred interactions and experiences with nature, feelings of safety and other concerns, and at the same time encourage pro-environmental behavior. This can be combined with the expertise and experience of the HCP about their target group. Next, co-creation of customized nature activities with the patient could lead to greater engagement and motivation for visiting nature. Finally, the PCC framework could also support a CI-approach (see 4.1.) or be used as theoretical framework underlying the healthcare of the target group. Intervention Mapping suggests as well to rely on evidence-based frameworks when designing health interventions.

Overall, the use of this PCC framework could provide opportunities for the design and implementation quality of integrated NBIs. However, further research is needed to learn how a PCC framework could be applied in the design and implementation of NBI.

### 4.3 One Health perspective in the design and implementation of NBI

The One Health approach to healthcare promotes a holistic, integrative and transdisciplinary perspective to research, design and implementation of complex healthcare interventions (57). Several of these aspects were not found in the NBI-studies.

Firstly, despite the scientific evidence on biodiversity-health link (18, 28, 58–60), and the recommendation of the WHO to consider this link in public healthcare (61), none of the reviewed NBI-studies addressed the quality of biodiversity of their surrounding natural environment. More concrete, the focus in the NBI appears to be on human health rather than on restoring biodiversity. Given the evidence on the physical link between biodiversity and health, as also described in One Health, it would be valuable to include in NBI the aspect of restoring biodiversity when designing NBI. As such, taking care for biodiversity becomes integrated in the healthcare of the target group, in which the physical health benefits would be valorized. The fact that biodiversity restoration was not mentioned in any of the publications, suggests that the integrative aspect of restoring biodiversity in NBI is still an emerging field. Nevertheless, some studies on healing or therapeutic gardens have considered both perspectives (62–64). As the gardens were not located at the healthcare facility and conceived as separate initiatives, these studies were not included in our review. Nevertheless, the results and guidelines of these studies could be transferable to the ecological design and implementation of NBI at healthcare facilities.

Secondly, it would be of high value to assess how the key characteristics of nature settings in NBIs are linked to variable human health outcomes. An emerging link between ecosystem and human health is postulated by the biodiversity hypothesis, which states that ‘contact with natural environments enriches the human microbiome, promotes immune balance and protects from allergy and inflammatory disorders’ (65). Small-scale studies indeed point to the transfer of environmental microorganisms to humans after urban green space exposure (66), however this was not explored in any of the reviewed studies, while it could serve as a quantitative quality outcome of contact with nature.

Thirdly, while in One Health, Planetary health and several studies (3, 67–70) a plea for improving a deeper, relational attitude toward nature, often referred to as ‘nature connectedness’, is made, the activities in the NBIs in this study were mainly focused on a unidirectional, instrumental use of nature to improve human health. There were no clear examples found how developing nature connectedness was a mean to improve wellbeing and a caring attitude toward nature. Two studies in this scoping review refer to ‘connection with nature’ (41) or ‘a close relationship with nature’ (52), however, the link with a reciprocal, relational attitude toward nature was missing. Moreover, as nature contact and nature connectedness are often used as synonym, while there is clearly a difference (68) the specificity of the nature interaction is not always clear. In addition, there is no clear indication in the selected studies concerning horticultural therapy of whether it focuses on organic gardening, how the horticulture garden is designed in a way that biodiversity can flourish or is cultivated in a reciprocal way with nature. In line with the IPBES report, an NBI can also be designed in such a way that consciously considers not only the instrumental values that nature offers, but also the intrinsic and relational values of nature (71). For example, a recent large-scale study shows how primary healthcare patients were taken into a highly biodiverse forest and were guided in sensorial exercises, with a positive impact on sleep duration and enhanced feeling of nature connectedness (72).

Fourth, One Health (61), and the frameworks of IM (25) and CI (24) promote a transdisciplinary and multidisciplinary approach to create leverage in all the layers of the organization and stakeholders in and outside the organization. Some studies suggested the presence of a multidisciplinary project group; however, nothing was said about a transdisciplinary, multilayered approach, in which all stakeholders of all layers of the organization are involved. Further research is required to get more insight in how a trans- and multidisciplinary and layered approach may contribute to the desired NBI-outcomes.

### 4.4 Quality assessment of health outcomes

To assess NBI effects on human health, it is highly important to measure and report key health-related outcomes. The studies in this review mostly focused on mental health outcomes that were measured with qualitative, mixed or (less frequently) quantitative methods. The reporting of specific quantitative physical health outcomes was limited, despite their high relevance especially in healthcare settings. For example, a recent systematic review reported distinct immunological benefits at the level of anti-inflammatory and anti-allergic markers from nature exposure such as forest bathing (73). Similar physical health-related outcomes and beneficial NBI-related mechanisms of action, its duration, frequency and intensity, should be explored to improve NBI quality in health care settings.

### 4.5 Ensuring sustainability in the design of nature-based interventions

From the perspective of Planetary Health, both in the structural design of the natural environment and in the organizational components of the NBI, it is important to ensure sustainability by respecting planetary boundaries. This means that the design and materials used do not contribute to additional greenhouse gas emissions and the further destruction of ecosystems worldwide. Whenever possible, materials and interventions that contribute to climate mitigation and climate adaptation as well as strengthening of local ecosystems should be selected (74, 75). However, this aspect was not considered in the selected studies.

### 4.6 Implications for practice

Our study showed that designing an NBI is a complex challenge, with several quality criteria such as biodiversity restoration, human health, intervention criteria and their interactions should be considered. Some implications for practice will be discussed in what follows.

First, given the evidence on the biodiversity-health link (17, 18, 58, 65, 76, 77), it is advisable to include in NBIs the role of restoring biodiversity in the surrounding natural environment of the healthcare facility. Therefore, a healthcare facility should conduct an environmental analysis of opportunities to bring biodiversity into its outdoor environment. If necessary, for example due to a lack of internal expertise, external experts should be consulted.

Second, the application of the PCC framework could enrich the design and implementation of NBIs (55). For example, the PCC framework promotes cultivating communication with all levels of the organization regarding the intervention, which consists of listening to the stakeholders about their needs, wishes and uncertainties, and inviting them to co-create the NBI. Next, training the HCPs about the possibilities and knowledge of NBI and sharing information with the patient about the possible positive effects and risks, could help patients, if possible, decide for themselves, whether and how to participate in a nature visit, or want to participate in work in the garden (e.g., horticultural therapy), and thereby build a partnership with the patient. Another example is that HCPs and those responsible for biodiversity could also learn from each other by sharing experiences and practices and adjust the intervention as necessary following new insights. In addition to the quality criteria proposed in this study, several elements of the PCC framework could also be considered to ensure person-centered care.

Finally, to come to integrated nature-based interventions, that take into account the biodiversity-health link, professionals involved in the NBI should be educated about the health benefits for humans and nature. Additionally, they could be trained in the One Health or Planetary Health frameworks. Although both have differences in approach, they both focus on the link between humans and nature and offer pathways for working with the complexities of designing and implementing integrated NBIs. Planetary health education is imperative for professionals dealing with intersecting challenges such as biodiversity loss and human health (78, 79).

### 4.7 Strengths and limitations of this study

To our knowledge, this scoping review is the first in the field on NBIs in healthcare facilities that combine biodiversity and the guidance in nature of their target group. This scoping review was conducted according to the JBI methodology for scoping reviews (33) and the PRISMA-SCR (34). We conducted a thorough inclusion and exclusion process involving two researchers and an interdisciplinary research team, the project steering group, and a panel of stakeholders. Nevertheless, this study has some limitations.

First, due to the time constraints of this study and the inclusion of English language publications, it is possible that certain studies may not have been identified. Second, research on NBIs is relatively new and has inconsistencies in scientific NBI terminology. Therefore, achieving consistency in the most relevant search terms to cover all available literature is a major challenge. Finally, we recognize that extending the review to NBIs outside of healthcare facilities could provide additional insight into NBI quality criteria.

### 4.8 Recommendations for research

With this study we have taken the first steps toward a preliminary NBI quality framework, which should be further tested and refined in the daily practice of healthcare facilities. Greater insight into these quality criteria will advance evidence-based research and provide policy makers and health professional education with a framework for robust design, implementation, and evaluation of NBI in healthcare facilities. In addition, it could also make a positive contribution to the continuity of NBI and the integration of the link between biodiversity and health in healthcare.

To test the validity of our set of quality criteria it would be advisable to conduct a field-based study in healthcare facilities to find out which quality criteria and how they are used in practice in the design and implementation of integrated NBIs, in which the biodiversity-health link is included. In addition, it would be valuable to better understand how the quality criteria interact with each other. In addition, further research is recommended that explores additional quality criteria underlying NBIs conducted outside of healthcare facilities and may be transferable to healthcare contexts. Furthermore, the integration of the biodiversity-health link into NBIs in healthcare facilities and how each identified quality criterion can be tailored to the respective target group should be studied in more detail. However, achieving standardization of NBIs appears to be challenging due to various contextual differences and individual specificity (80).

## Author contributions

AS: Conceptualization, Methodology, Writing – original draft. BD: Conceptualization, Methodology, Writing – review & editing. GB: Writing – review & editing. IS: Writing – review & editing. RS: Writing – review & editing. RR: Supervision, Writing – review & editing, Conceptualization, Methodology. HK: Supervision, Writing – review & editing, Conceptualization, Methodology.

## Footnotes

^1^ https://www.planetaryhealthalliance.org/clinicians-for-planetary-health

^2^ https://www.climatepsychologyalliance.org/
